# Genome-Wide Discovery and Evaluation of Kazakh Ancestry-Informative Markers

**DOI:** 10.3390/life16101652

**Published:** 2026-09-30

**Authors:** Zhassulan Zhaniyazov, Akmaral Kulatayeva, Nazym Altynova, Zhuldyz Zhaksybay, Assel Baidullayeva, Aikorkem Mustafayeva, Olzhas Iksan, Rozaimi Razali, Leyla Djansugurova

**Affiliations:** 1 Institute of Genetics and Physiology, CS MSHE RK, Almaty 050060, Kazakhstanleylad@mail.ru (L.D.); 2 Faculty of Biology and Biotechnology, Al-Farabi Kazakh National University, Almaty 050040, Kazakhstan; 3 K. Turysov Institute of Geology and Oil and Gas Engineering, Satbayev University, Almaty 050013, Kazakhstan; 4 Department of Biomedical Sciences, Qatar University, Doha 2713, Qatar; rozaimi.r@qu.edu.qa

**Keywords:** Kazakh population, ancestry-informative markers, Central Asia, genetic ancestry, population genetics, single-nucleotide polymorphisms, transferability assessment, nearest-centroid assignment

## Abstract

Kazakh populations occupy an important position within the genetic landscape of Central Asia and Inner Eurasia, yet compact ancestry-informative marker panels evaluated for Kazakh-related ancestry remain limited. We analyzed genome-wide single-nucleotide polymorphism data from 624 Kazakh individuals together with data from external reference populations. After harmonization, the discovery dataset comprised 1607 individuals from 41 population groups and 80,766 shared autosomal variants. Candidate ancestry-informative markers were ranked using allele-frequency differentiation and informativeness for assignment, followed by linkage disequilibrium filtering. The final panel comprised 385 markers. Because marker availability differed across external datasets, transferability assessment in two independent Kazakh cohorts (*n* = 224 and *n* = 132) was performed using the 341-marker subset shared across the internal dataset and both external cohorts. Principal component analysis showed that both external Kazakh cohorts overlapped substantially with the internal Kazakh cluster. In nearest-centroid analysis, 94.6% of internal Kazakh individuals and 92.0% and 91.7% of individuals from the two external Kazakh cohorts, respectively, were assigned as Kazakh-related. Among non-Kazakh reference individuals used in the discovery framework, 4.1% were assigned as Kazakh-related; this internal, selection-informed estimate was mainly driven by genetically close Central Asian and Inner Eurasian populations. These findings support the transferability of the shared marker subset across independent Kazakh datasets, while also showing limited discrimination between Kazakhs and several closely related neighboring populations.

## 1. Introduction

Central Asia and Inner Eurasia represent one of the major contact zones in human population history, where western Eurasian, eastern Eurasian, Siberian, and steppe-related ancestries have interacted through repeated population movements and admixture events. These processes contributed to the complex genetic structure of present-day Eurasian populations, including Turkic-speaking groups whose genetic profiles reflect both local population histories and historical gene flow across the steppe belt [1,2,3,4].

Kazakh populations occupy an important position within this genetic landscape. Previous genome-wide studies have placed Kazakhs within the broader Central Asian and Inner Eurasian genetic continuum and have demonstrated admixed genetic profiles reflecting both East Eurasian and West Eurasian ancestry components [5,6,7,8]. A recent large-scale genotyping study has further expanded the genomic resources available for the Kazakh population [9]. These studies provide an important genomic background, but compact ancestry-informative marker panels specifically evaluated for Kazakh-related ancestry remain limited.

Ancestry-informative markers (AIMs) are genetic variants that show substantial allele-frequency differences among populations and can be used to infer ancestry within a defined reference framework. AIMs can be selected using different approaches, including allele-frequency differentiation, pairwise F_ST_ ranking, informativeness for assignment, and PCA-related marker prioritization [10,11,12,13,14,15]. Previous studies have shown that carefully selected AIM panels can capture ancestry-related population structure using substantially fewer markers than genome-wide single-nucleotide polymorphism (SNP) datasets [13,14,15].

For populations with complex admixture histories, AIM selection requires careful interpretation because ancestry patterns may be continuous and partially shared with neighboring populations. This issue is particularly relevant for Kazakhs and other Central Asian populations, where genetic differentiation from related Inner Eurasian groups may be modest [5,6,7,8,9]. Therefore, a Kazakh AIM panel should be developed within a clearly defined reference-population framework and evaluated in independent Kazakh datasets rather than interpreted as a marker set unique to Kazakh ethnicity.

The aim of the present study was to identify a compact set of SNPs informative for Kazakh-related ancestry and to evaluate the transferability of the shared marker subset in independent Kazakh datasets. We analyzed genome-wide SNP data from 624 Kazakh individuals together with data from external global and Eurasian reference populations. Candidate AIMs were ranked using allele-frequency differentiation and informativeness for assignment, followed by linkage disequilibrium (LD) filtering and principal component analysis (PCA) loading characterization. The final LD-filtered panel consisted of 385 SNPs. We then evaluated the full panel internally within the discovery framework and assessed the transferability of the 341-SNP subset shared with two independent Kazakh cohorts from Kazakhstan and China. The study was designed to develop a Kazakh ancestry-informative marker resource within a defined reference-population context, rather than a test of ethnic identity or categorical population identity.

## 2. Materials and Methods

### 2.1. Study Cohort and Genotype Data

The internal study cohort comprised 624 adult individuals who self-identified as Kazakh and were residents of Kazakhstan. DNA samples were available through the biobank of the Institute of Genetics and Physiology. All participants provided written informed consent before biological sample collection. Detailed regional information was not included in the present analysis. Therefore, the cohort was analyzed as a single Kazakh target population because the study aimed to identify markers informative for Kazakh-related ancestry at the population level rather than to investigate regional, tribal, or subethnic structure within Kazakhs.

DNA samples were genotyped using the Illumina Global Screening Array v3 platform (Illumina Inc., San Diego, CA, USA). Although the samples were processed in different genotyping batches, the same array platform was used throughout the internal cohort. A separate batch-stratified QC analysis was not performed, which is acknowledged as a limitation of the present study. Genomic coordinates were based on the GRCh38 reference genome. Genotype data were converted to PLINK binary format, and the analysis was restricted to autosomal SNPs.

The dataset used for AIM analysis included 624 individuals. Sample-level genotype missingness was assessed using a minimum call-rate threshold of 95%, and all 624 individuals passed this criterion and were retained for downstream analyses. Duplicate samples and closely related individuals were identified using identity-by-descent estimates in PLINK, with PI_HAT > 0.125 used as the relatedness threshold. No duplicate or closely related individuals exceeding this threshold were detected.

Sample-level heterozygosity was additionally evaluated using the PLINK method-of-moments inbreeding coefficient (F). For this diagnostic analysis only, autosomal variants with genotype missingness > 2% or minor allele frequency (MAF) < 0.05 were excluded, followed by LD pruning using a 200-variant window, a 50-variant step size, and an r^2^ threshold of 0.2. This resulted in 100,016 LD-pruned autosomal SNPs for heterozygosity assessment. Individuals with F values outside the cohort mean ± 3 standard deviations were flagged for further inspection. Six individuals exceeded this threshold, all in the direction of increased homozygosity. Their genotype missingness across the same 100,016-SNP marker set was low (0.018–0.176%), and all had passed the predefined sample-missingness and relatedness criteria. These individuals were therefore retained, as there was no indication that their heterozygosity deviation was associated with poor genotype completeness.

Hardy–Weinberg equilibrium (HWE) and MAF thresholds were not used as variant-exclusion criteria during AIM discovery. This was intentional because the objective was to identify ancestry-informative allele-frequency differences rather than to perform genotype–phenotype association testing. Deviations from HWE may arise from genuine population structure, admixture, or non-random mating, while application of a global MAF threshold could remove variants that are relatively uncommon within one population but show substantial allele-frequency differentiation between populations. HWE- and MAF-based filtering was therefore omitted at the AIM-discovery stage to preserve potentially informative population-specific variation. The MAF threshold described above was used exclusively for the heterozygosity diagnostic and was not applied to AIM candidate selection.

No individuals were excluded solely based on their position in ancestry analyses. This was consistent with the objective of evaluating Kazakh-related ancestry variation within the available cohort rather than defining a more genetically homogeneous subset.

Before merging with the external reference datasets, SNP identifiers, genomic coordinates, and allele coding were harmonized across datasets. Strand-ambiguous, multiallelic, duplicated, non-autosomal, and unresolved variants were removed during cross-dataset matching. Only shared autosomal SNPs with consistent allele coding were retained for downstream analyses.

The study was approved by the Local Ethics Committee of the Institute of Genetics and Physiology, Committee of Science, Ministry of Science and Higher Education of the Republic of Kazakhstan (Protocol No. 3 dated 30 October 2023). All procedures were performed in accordance with relevant guidelines and regulations. All participants provided written informed consent for participation in the study and for the use of their biological samples and genetic data for research purposes prior to biological sample collection.

### 2.2. Reference Datasets and Dataset Harmonization

External reference populations included selected populations from the 1000 Genomes Project and the Estonian Biocentre Human Genome Diversity Panel (EGDP) [16,17]. The 1000 Genomes Project reference populations were obtained from the Phase 3 dataset distributed through the PLINK 2.0 Resources repository. The EGDP reference data were obtained from the Estonian Biocentre public data resource associated with Pagani et al. [17]. Together, these datasets provided global and Eurasian reference populations for Kazakh AIM discovery.

All datasets were harmonized to the GRCh38 genome build before merging. The internal Kazakh dataset was already based on GRCh38 coordinates, whereas the EGDP reference dataset was originally aligned to GRCh37. Before coordinate conversion, reference-allele consistency was checked against the GRCh37 reference genome using BCFtools +fixref v1.24. EGDP variants were then converted to GRCh38 using Picard LiftoverVcf v3.3.0 with the hg19ToHg38.over.chain file and the GRCh38 reference genome. After coordinate conversion, variants were matched across datasets using SNP identifiers and GRCh38 genomic positions, and allele coding was checked for concordance. Non-autosomal, multiallelic, duplicated, strand-ambiguous, and variants with unresolved allele discrepancies were excluded. Only shared autosomal SNPs with concordant allele coding were retained for downstream analyses.

The internal Kazakh dataset contained 603,354 markers, whereas the preprocessed EGDP dataset used for merging contained 300,301 markers. Cross-dataset matching identified 80,766 markers shared between the Kazakh and EGDP datasets. The selected 1000 Genomes Phase 3 reference samples were subsequently extracted at these same 80,766 marker positions before merging. Consequently, no further reduction in marker number occurred during the 1000 Genomes merge step. The final harmonized discovery dataset therefore comprised 1607 individuals from 41 population groups and 80,766 shared autosomal SNPs, including 624 Kazakh individuals, 433 EGDP individuals, and 550 individuals from selected 1000 Genomes populations. A stepwise summary of marker retention during dataset harmonization is provided in Supplementary Table S12.

### 2.3. Population Structure Analysis

Principal component analysis (PCA) was performed to evaluate the position of the Kazakh cohort relative to external reference populations. PCA was conducted in PLINK v1.9.0-b.8 using LD-pruned autosomal SNPs [18,19]. LD pruning was performed using a sliding window of 200 SNPs, a step size of 50 SNPs, and an r^2^ threshold of 0.2.

Population structure was examined at three levels: a global dataset including all selected reference populations, a Eurasian dataset excluding African and American outgroups, and a fine-scale regional dataset consisting primarily of Central Asian, Siberian, Turkic-speaking, Mongolic-speaking, and neighboring Eurasian populations. Principal components were visualized according to population and reference region. The percentage of variance explained by each principal component was calculated by dividing its eigenvalue by the sum of all eigenvalues from the corresponding PCA. The calculations were based on 1606 eigenvalues for the global PCA, 1406 for the Eurasian PCA, and 942 for the fine-scale regional PCA. For the subsequent AIM-based analyses, 385 eigenvalues were used for the internal 385-SNP PCA and 341 for the combined transferability PCA based on the shared 341-SNP subset.

### 2.4. Pairwise F_ST_ Analysis

Pairwise genetic differentiation between the Kazakh cohort and each reference population was estimated using the Weir–Cockerham F_ST_ estimator [20], as implemented in PLINK v2.0.0-a.7.4 M1 [19]. F_ST_ values were calculated using LD-pruned autosomal SNPs for the global, Eurasian, and fine-scale regional datasets. LD pruning retained 52,138 SNPs in the global dataset (*n* = 1607), 50,797 SNPs in the Eurasian dataset (*n* = 1407), and 50,609 SNPs in the fine-scale regional dataset (*n* = 943). Complete pairwise F_ST_ results for the global, Eurasian, and fine-scale analyses are provided in Supplementary Tables S2–S4, respectively. F_ST_ values were used descriptively to characterize relative genetic differentiation within the defined reference framework rather than for formal hypothesis testing; therefore, resampling-based confidence intervals were not calculated. Very low F_ST_ values for closely related populations were interpreted as evidence of limited differentiation rather than categorical population separation.

### 2.5. Candidate AIM Discovery and Ranking

Candidate AIMs were identified using the fine-scale dataset comprising the Kazakh cohort and 16 reference populations with at least 10 individuals: Karakalpak, Kyrgyz, Kalmyk, Altai, Mongol, Hakas, Bashkir, Tuva, Buriat, Turkmen, Nogay, Selkup, Tatar, Tajik, Evenk, and Kumyk. These populations were selected from the fine-scale regional reference set to represent genetically and geographically relevant Central Asian and neighboring Inner Eurasian populations. For each SNP, absolute allele-frequency differences (ΔAF) between the Kazakh cohort and each reference population were calculated. To prioritize markers showing consistent differentiation across populations, the differentiation score combined the mean absolute allele-frequency difference with its 10th percentile:

D = mean(|ΔAF|) + Q10(|ΔAF|).(1)

Informativeness for assignment (In) was calculated according to Rosenberg et al. [11], with each population contributing equally regardless of sample size. Because D and In were measured on different scales, both were min–max normalized to a 0–1 range and combined with equal weights:

Composite AIM score = scaled D + scaled In.(2)

Because both components were independently min–max scaled to the interval [0,1], their unweighted sum gives equal weight to differentiation and informativeness. During AIM ranking in the harmonized dataset, candidate SNPs were required to have a call rate of at least 95% in the Kazakh cohort and 90% in each reference population. Variants meeting these criteria were ranked by the composite AIM score, and the top 500 SNPs were carried forward for LD filtering.

### 2.6. PCA Loading Support and LD Filtering

The 500 highest-ranked candidate SNPs were evaluated for linkage disequilibrium in the pooled combined Kazakh and reference dataset of 1558 individuals using PLINK v1.9.0-b.8 [18,19]. Pairwise r^2^ was calculated within a 1 Mb window. SNPs were considered in descending order of their composite AIM score, and lower-ranked variants with r^2^ ≥ 0.20 with a previously retained SNP were excluded. This procedure resulted in a final panel of 385 LD-pruned AIMs. For each of the 115 excluded SNPs, the corresponding retained marker, pairwise r^2^ value, and genomic distance were recorded.

PCA loadings were calculated for the retained markers to characterize their contribution to the main axes of population structure. These loadings were used only for descriptive characterization and did not influence marker selection.

The robustness of the AIM-selection procedure was evaluated under several alternative analytical settings, including changes in the relative weights assigned to differentiation and informativeness, removal of the Q_10_ component, variation in candidate-pool size and LD threshold, and LD estimation using only the 624 Kazakh individuals. The final panel was also compared with 100 chromosome-matched random LD-pruned panels. In addition, a reduced panel based on the top 200 ranked candidates was evaluated using the same nearest-centroid framework.

### 2.7. Internal Evaluation of the Final AIM Panel

The final 385-SNP AIM panel was evaluated within the discovery dataset to determine whether it captured the main Kazakh-related population structure observed in the genome-wide analyses. Genotype data for the 385 AIMs were extracted from the combined Kazakh and reference dataset, and PCA was performed using only these markers.

This analysis was considered an internal evaluation of the final marker set rather than an independent validation, because the same Kazakh and reference samples had been used during AIM discovery. The purpose of this step was to assess whether the LD-filtered 385-SNP panel reproduced the main population-structure patterns before evaluating its transferability in independent external Kazakh cohorts.

### 2.8. Transferability Assessment Using Independent Kazakh Cohorts

Transferability was assessed using two independent Kazakh cohorts that were not used during AIM discovery or construction of the nearest-centroid assignment framework. The first cohort included 224 Kazakh individuals reported by Serikzhan et al. [9]; processed VCF files were obtained from the European Variation Archive under accession PRJEB89820. The second cohort was obtained through controlled access from the Genome Variation Map resource associated with Lei et al. [6] under project accession GVM000484. The downloaded Illumina Zhonghua genotype dataset initially included 133 individuals. After LD pruning, pairwise relatedness was assessed in PLINK v1.9 using a PI_HAT threshold of 0.125. One pair exceeded this threshold (PI_HAT = 0.129), and the individual with the higher genotype missingness was excluded, resulting in a final cohort of 132 individuals.

Because both external datasets consisted of Kazakh individuals, they were used to assess transferability of the AIM panel rather than specificity against non-Kazakh populations.

Marker overlap was evaluated after harmonization of SNP identifiers, genomic coordinates, and allele coding with the final AIM panel. For the Serikzhan et al. dataset, 372 of the 385 AIMs were available, whereas 353 AIMs were available in the Lei et al. dataset. A common subset of 341 AIMs was shared across the internal combined Kazakh and reference dataset and both external cohorts and was therefore used for the combined transferability analyses. The complete 385-SNP panel could not be evaluated in both external datasets because marker availability differed across genotyping platforms.

As a post-harmonization quality check, allele frequencies of the 341 shared AIMs were compared between the internal Kazakh cohort and each external cohort to identify potential allele-orientation inconsistencies. Allele coding was concordant for all shared markers. Mean sample-level genotype missingness was 0.136% in the Serikzhan et al. cohort and 0.124% in the Lei et al. cohort, with maximum values of 3.23% and 1.17%, respectively. No individual in either cohort had genotype missingness above 5%. These checks were used to assess the consistency of the shared marker set across datasets generated on different platforms and batches.

### 2.9. Nearest-Centroid Assignment Framework

Nearest-centroid assignment analysis was performed using the 341 AIMs shared across the internal combined Kazakh and reference dataset and both external Kazakh cohorts. The internal reference framework included 624 Kazakh individuals and 934 individuals from 32 non-Kazakh reference populations. Reference groups represented by fewer than 10 individuals were excluded from this analysis, resulting in a classification dataset of 1558 individuals.

Genotypes were represented as allele dosages (0, 1, or 2), and missing values were imputed using SNP-specific means calculated from the internal combined Kazakh and reference dataset. No additional scaling was applied. The external Serikzhan et al. and Lei et al. cohorts were kept independent from construction of the assignment framework and were not used to estimate missing values, calculate centroids, or define the assignment rule.

For each SNP, a Kazakh centroid was calculated from the internal Kazakh cohort, and separate centroids were calculated for each non-Kazakh reference population. Euclidean distance was then calculated from each individual to the Kazakh centroid and to each non-Kazakh centroid. An individual was assigned as Kazakh-related when the Kazakh centroid was closer than the nearest non-Kazakh centroid.

For internal Kazakh individuals, the Kazakh centroid was recalculated after excluding the individual being evaluated. The same leave-one-out procedure was applied to non-Kazakh reference individuals with respect to their own population centroid. Full internal-reference centroids were used for the two external Kazakh cohorts.

Assignment rates were reported as n/N and percentages with 95% Wilson confidence intervals. The reported 95% Wilson confidence intervals quantify binomial uncertainty in the observed assignment proportions conditional on the fixed marker panel and assignment rule; they do not incorporate uncertainty arising from upstream marker selection or centroid development. A distance margin was also calculated as the distance to the nearest non-Kazakh centroid minus the distance to the Kazakh centroid, with positive values indicating closer proximity to the Kazakh centroid. Because the non-Kazakh reference individuals were part of the discovery/reference framework, their assignment rate was interpreted as an internal, selection-informed estimate rather than as an independent external specificity estimate.

Analyses were performed using PLINK v1.9.0-b.8 and PLINK v2.0.0-a.7.4 M1 together with custom Python 3.12 scripts. Detailed software versions, analysis scripts, command-line workflows, and non-identifying derived data are provided in Supplementary File S1.

## 3. Results

### 3.1. Dataset Composition and Population Structure

After genotype harmonization and merging with the external reference datasets, the final discovery dataset comprised 1607 individuals from 41 population groups and 80,766 shared autosomal SNPs. These included 624 Kazakh individuals, 433 individuals from the Estonian Biocentre Human Genome Diversity Panel, and 550 individuals from selected 1000 Genomes Project populations (Table 1). The reference panel included populations from Central Asia, Siberia, the Volga–Ural region, West Eurasia, East Asia, South Asia, Africa, and the Americas. The complete population-level composition of the discovery dataset is provided in Supplementary Table S1.

**Figure 1:**
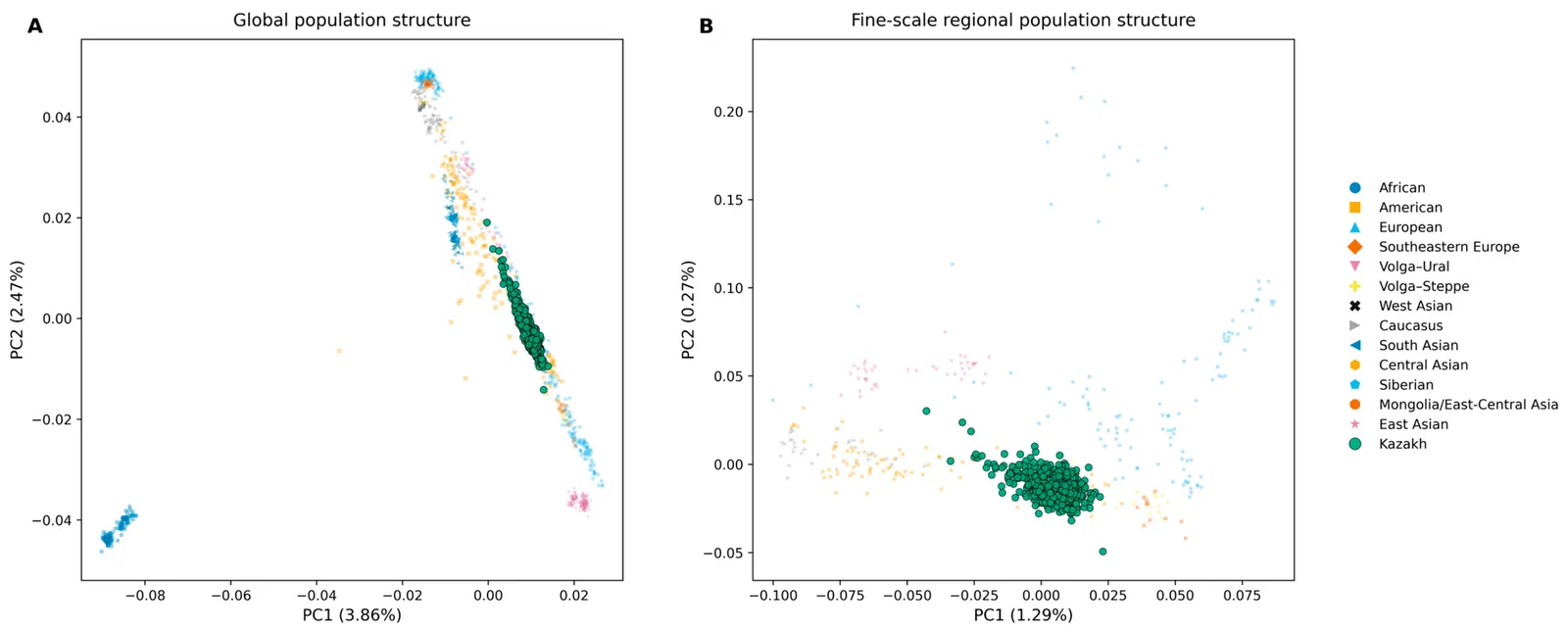
Principal component analysis of the Kazakh cohort and external reference populations. (**A**) Global PCA showing the position of Kazakh individuals within worldwide population variation. (**B**) Fine-scale regional PCA showing the position of the Kazakh cohort relative to Central Asian, Siberian, Volga–Ural, Volga–Steppe, Mongolian/East-Central Asian, Caucasus, and neighboring Eurasian populations. Kazakh individuals are highlighted in green. Percentages shown on the axes indicate the proportion of total variance explained by each principal component, calculated using all available eigenvalues.

Principal component analysis showed the position of the Kazakh cohort within the global and regional reference structure (Figure 1). In the global PCA, African reference populations formed a distinct cluster, whereas Eurasian populations were distributed along a broad west–east axis of genetic variation. Kazakh individuals occupied an intermediate position along this axis, between West Eurasian and East Eurasian reference groups (Figure 1A).

The Eurasian PCA showed a similar intermediate position of the Kazakh cohort (Figure S1). In the fine-scale regional PCA, Kazakh individuals formed a dense central cluster between West and East Inner Eurasian reference populations (Figure 1B). Most Kazakh individuals clustered closely together, although a small number showed greater dispersion within the regional genetic space. Overall, these patterns were consistent with analyzing the 624 Kazakh individuals as a single target cohort for subsequent AIM discovery.

### 3.2. Genetic Differentiation Between Kazakhs and Reference Populations

**Figure 2:**
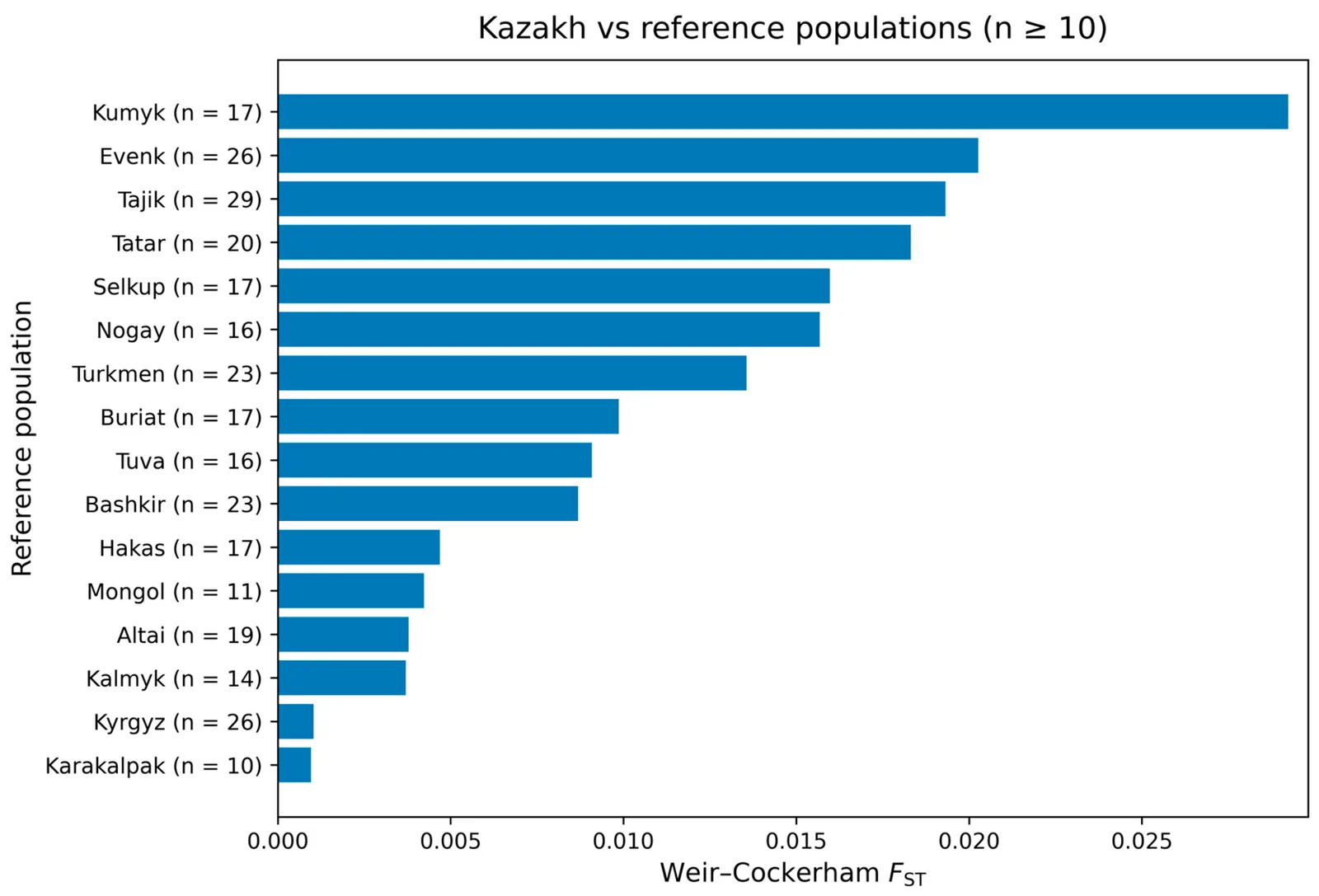
Pairwise Weir–Cockerham F_ST_ values between the Kazakh cohort (*n* = 624) and fine-scale reference populations represented by at least 10 individuals, calculated using 50,609 LD-pruned autosomal SNPs. Reference-population sample sizes were: Karakalpak, *n* = 10; Kyrgyz, *n* = 26; Kalmyk, *n* = 14; Altai, *n* = 19; Mongol, *n* = 11; Hakas, *n* = 17; Bashkir, *n* = 23; Tuva, *n* = 16; Buriat, *n* = 17; Turkmen, *n* = 23; Nogay, *n* = 16; Selkup, *n* = 17; Tatar, *n* = 20; Tajik, *n* = 29; Evenk, *n* = 26; and Kumyk, *n* = 17. Lower F_ST_ values indicate lower genetic differentiation from the Kazakh cohort. Complete results for all evaluated fine-scale reference populations are provided in Supplementary Table S4.

Pairwise Weir–Cockerham F_ST_ estimates were used to assess genetic differentiation between the Kazakh cohort and the selected reference populations. Complete results for the global, Eurasian, and fine-scale analyses are provided in Supplementary Tables S2–S4, respectively. The main fine-scale comparison included only populations represented by at least 10 individuals (Figure 2; Supplementary Table S5).

The lowest levels of differentiation were observed between the Kazakh cohort and the Karakalpak and Kyrgyz populations (F_ST_ = 0.0010 for both), followed by Kalmyk (F_ST_ = 0.0037), Altai (F_ST_ = 0.0038), Mongol (F_ST_ = 0.0042), and Hakas (F_ST_ = 0.0047). These results were consistent with the PCA analyses, which placed the Kazakh cohort close to several Central Asian and Inner Eurasian populations.

Intermediate F_ST_ values were observed for Bashkir, Tuva, and Buriat populations (0.0087–0.0099), whereas higher values were observed for Turkmen, Nogay, Selkup, Tatar, Tajik, Evenk, and Kumyk populations (0.0136–0.0292). Among the fine-scale populations included in the main analysis, Kumyk showed the highest differentiation from the Kazakh cohort.

Overall, the F_ST_ results indicated that the Kazakh cohort was genetically closest to several neighboring Central Asian and Inner Eurasian populations within the tested reference framework. The very low F_ST_ values observed for the Karakalpak and Kyrgyz populations indicate limited genetic differentiation and should not be interpreted as evidence of categorical population separation. These estimates were used descriptively to characterize relative genetic differentiation and to provide population-genetic context for subsequent AIM discovery.

### 3.3. Discovery of the Final 385-SNP Kazakh AIM Panel

Application of the composite AIM-ranking procedure identified the 500 highest-ranked QC-passing candidate SNPs. After LD filtering, 385 LD-pruned SNPs were retained and constituted the final Kazakh AIM panel.

The retained markers were distributed across all autosomes, showing broad genomic coverage rather than concentration within a single chromosomal region. PCA loading scores were calculated for descriptive characterization of the retained markers. The complete list of 385 AIMs, including genomic coordinates, allele information, allele-frequency differentiation metrics, informativeness-for-assignment values, composite AIM scores, PCA loading scores, and original composite-score ranks, is provided in Supplementary Table S6.

The final 385-SNP panel was subsequently evaluated within the internal dataset and tested for transferability in the two independent external Kazakh cohorts.

#### Robustness of AIM Selection

Robustness analyses showed that the selected panel remained largely stable across alternative analytical settings. When LD was re-estimated using only the 624 Kazakh individuals, 383 of the original 385 SNPs were retained (99.5%; Jaccard similarity = 0.977). Moderate changes in the relative weighting of differentiation and informativeness retained 321 of the original markers (83.4%), whereas differentiation-only and informativeness-only rankings retained 250 and 273 markers, respectively. Removal of the Q10 component retained 309 of the original 385 markers (80.3%).

Changing the LD threshold primarily affected panel size. A more stringent threshold (r^2^ = 0.10) produced a subset of 356 SNPs, whereas thresholds of 0.30 and 0.50 retained the original 385 markers together with additional variants. No SNP pair in the final panel had residual r^2^ ≥ 0.20 within 1 Mb, with a maximum observed r^2^ of 0.199.

The final 385-SNP panel showed higher marker-level differentiation and informativeness than all 100 chromosome-matched random LD-pruned panels across the six evaluated marker-level metrics (empirical one-sided *p* = 0.0099 for each comparison; Supplementary Table S9). This benchmark was used to assess marker informativeness and was not interpreted as an independent evaluation of external classification performance. Reducing the candidate panel to the top 200 markers also decreased assignment performance: the resulting panel contained 180 markers shared across all datasets and produced Kazakh-related assignment rates of 86.1% in the internal Kazakh cohort, 80.8% in the Serikzhan cohort, and 80.3% in the Lei cohort, compared with 94.6%, 92.0%, and 91.7%, respectively, for the 341-SNP common panel. Detailed robustness results are provided in Supplementary Tables S7–S9.

### 3.4. Evaluation and Transferability Assessment of the Final Kazakh AIM Panel

The final Kazakh AIM panel was evaluated in three stages. First, the complete 385-SNP panel was assessed within the discovery dataset to determine whether it preserved the main Kazakh-reference population structure. Second, transferability was evaluated in two independent Kazakh cohorts using the 341 AIMs shared across all datasets. Third, nearest-centroid assignment analysis was used to quantify Kazakh-related assignment rates in the internal and external Kazakh cohorts and to assess Kazakh-related assignments among the non-Kazakh reference individuals.

#### 3.4.1. Internal PCA Evaluation

**Figure 3:**
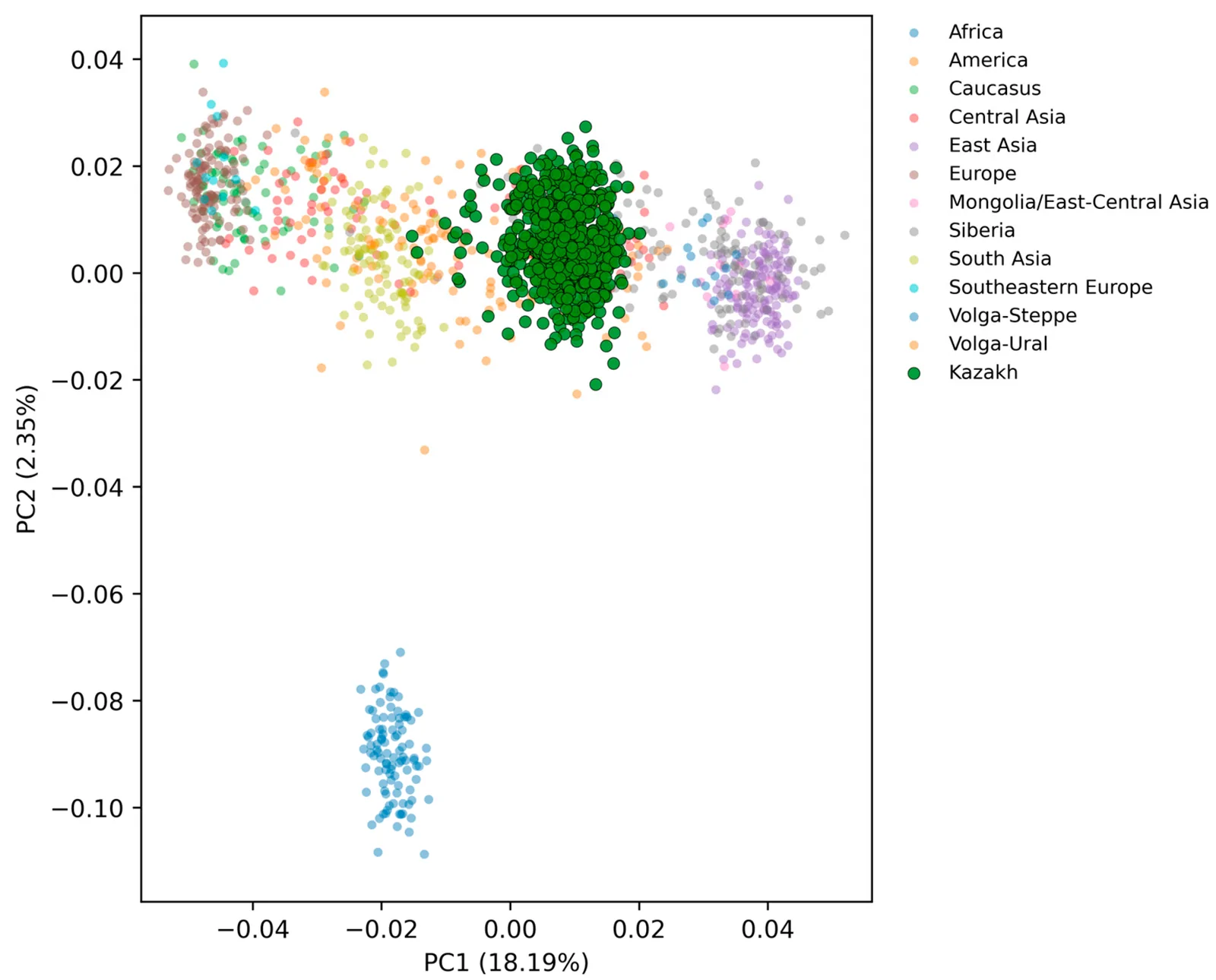
Internal PCA evaluation of the final 385-SNP Kazakh ancestry-informative marker panel. PCA was performed within the discovery dataset using only the 385 selected AIMs. Kazakh individuals formed a relatively compact cluster in an intermediate position between western and eastern Eurasian reference populations, with partial overlap with neighboring Central Asian and Inner Eurasian groups. PC1 and PC2 explained 18.19% and 2.35% of the total variation, respectively.

The final 385-SNP Kazakh AIM panel was first evaluated within the discovery dataset to determine whether the selected markers preserved the main population structure observed in the genome-wide analyses. Genotype data for the 385 AIMs were extracted from the combined Kazakh and reference dataset, and PCA was performed using only the selected markers (Figure 3).

The first two principal components explained 18.19% and 2.35% of the total genetic variation, respectively. Along PC1, West Eurasian reference populations were positioned toward negative values, whereas East Asian and Siberian populations were positioned toward positive values. Kazakh individuals formed a relatively compact cluster in an intermediate position between these western and eastern Eurasian groups. Partial overlap was observed with Central Asian and neighboring Inner Eurasian populations, while African reference individuals were clearly separated from the Eurasian populations along PC2.

These results showed that the final 385-SNP panel preserved the main broad-scale population structure observed in the discovery dataset despite representing only a small subset of the original SNP set. Because the same Kazakh and reference individuals were used for both marker discovery and PCA evaluation, this analysis was considered an internal assessment of population-structure preservation rather than an independent validation.

#### 3.4.2. Transferability Assessment in Independent Kazakh Cohorts

Transferability was evaluated in two independent Kazakh cohorts: 224 individuals from the Serikzhan et al. dataset [9] and 132 individuals from the Lei et al. dataset [6].

Of the final 385 AIMs, 372 were available in the Serikzhan et al. dataset and 353 in the Lei et al. dataset. A common set of 341 AIMs was shared across the combined Kazakh and reference dataset and both external Kazakh cohorts and was therefore used for the transferability analyses. Marker-overlap results are provided in Supplementary Table S10.

Allele frequencies of the 341 shared AIMs were highly concordant between the internal Kazakh cohort and the Serikzhan et al. cohort (Pearson r = 0.954; median |ΔAF| = 0.021; maximum = 0.083) and between the internal cohort and the Lei et al. cohort (r = 0.934; median |ΔAF| = 0.025; maximum = 0.144). Allele coding was concordant for all 341 markers, no marker showed |ΔAF| > 0.20, and no evidence of residual allele-orientation errors was detected.

**Figure 4:**
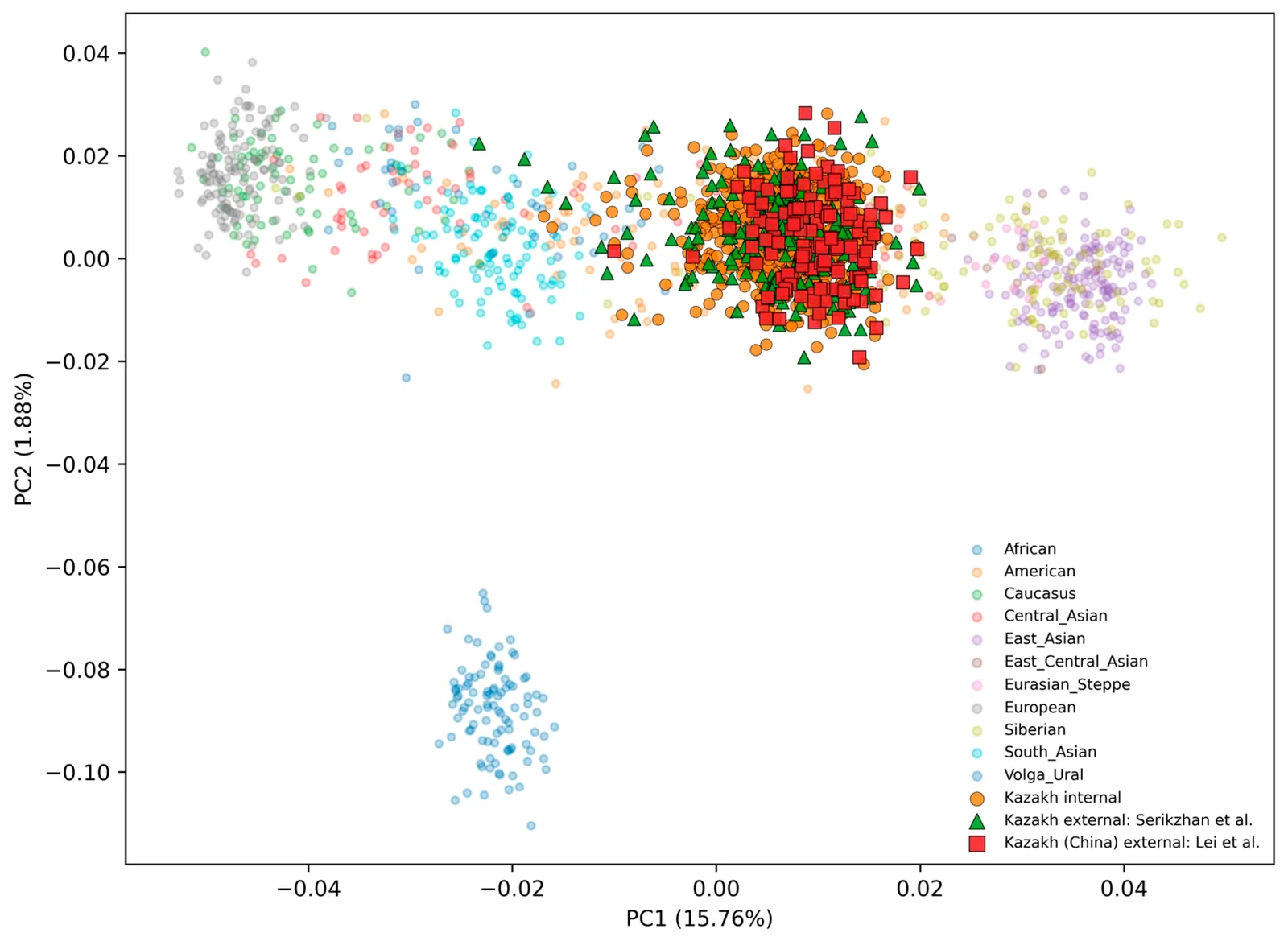
PCA-based transferability assessment of the Kazakh ancestry-informative marker subset in two independent Kazakh cohorts. PCA was performed using the 341 AIMs shared across the internal combined Kazakh and reference dataset, the Serikzhan et al. [9]. cohort, and the Lei et al. [6] cohort. Both external Kazakh cohorts overlapped substantially with the internal Kazakh cluster and occupied a similar position within the Central Asian and Inner Eurasian reference structure. Because the PCA axes were recomputed in the combined dataset, this analysis was interpreted as exploratory cross-dataset visualization. PC1 and PC2 explained 15.76% and 1.88% of the total variation, respectively.

The combined PCA dataset included 1914 individuals: 1558 from the internal Kazakh and reference dataset, 224 from the Serikzhan et al. cohort, and 132 from the Lei et al. cohort. PCA based on the 341 shared AIMs showed substantial overlap of both external Kazakh cohorts with the internal Kazakh cluster, with all three cohorts occupying a similar intermediate position within the Central Asian and Inner Eurasian reference structure (Figure 4). Because the PCA axes were recalculated using the combined dataset, this analysis was interpreted as an exploratory visualization of cross-dataset clustering rather than as an independent specificity assessment.

#### 3.4.3. Nearest-Centroid Assignment Analysis

Nearest-centroid assignment analysis was performed using the 341 AIMs shared across all datasets. Using this framework, 590 of 624 internal Kazakh individuals were assigned as Kazakh-related, corresponding to an assignment rate of 94.6% (95% CI: 92.5–96.1%; Table 2).

High Kazakh-related assignment rates were also observed in the two independent external cohorts. In the Serikzhan et al. cohort, 206 of 224 individuals were assigned as Kazakh-related, corresponding to 92.0% (95% CI: 87.7–94.9%). In the Lei et al. cohort, 121 of 132 individuals were assigned as Kazakh-related, corresponding to 91.7% (95% CI: 85.7–95.3%).

Among the 934 non-Kazakh reference individuals, 38 were assigned as Kazakh-related, corresponding to an internal, selection-informed assignment rate of 4.1% (95% CI: 3.0–5.5%). Because these individuals were part of the discovery and reference framework, this value should not be interpreted as an independent external estimate of specificity.

Kazakh-related assignments among non-Kazakh reference individuals were concentrated mainly in genetically close Central Asian and Inner Eurasian populations. The highest rates were observed in Karakalpak individuals (8/10, 80.0%; 95% CI: 49.0–94.3%) and Kyrgyz individuals (13/26, 50.0%; 95% CI: 32.1–67.9%), followed by Altai (5/19, 26.3%; 95% CI: 11.8–48.8%), Hakas (4/17, 23.5%; 95% CI: 9.6–47.3%), and Chukchi individuals (4/17, 23.5%; 95% CI: 9.6–47.3%). Single Kazakh-related assignments were also observed among Bashkir, Evenk, PEL, and MXL individuals. Population-level nearest-centroid results, including distance-margin summaries, are provided in Supplementary Table S11.

These findings were consistent with good transferability of the shared 341-SNP marker set to the two independent Kazakh cohorts. At the same time, the non-Kazakh assignment results showed limited discrimination from several genetically close neighboring populations, particularly Karakalpak and Kyrgyz groups.

## 4. Discussion

In this study, we developed and evaluated a compact Kazakh ancestry-informative marker panel based on genome-wide SNP data from 624 Kazakh individuals and a broad set of external reference populations. The final panel comprised 385 LD-pruned AIMs, of which 341 were shared across the discovery dataset and both independent external Kazakh cohorts. The shared marker set showed high Kazakh-related assignment rates in both external cohorts, while 4.1% of the non-Kazakh reference individuals were assigned as Kazakh-related. Because these non-Kazakh individuals were part of the discovery and reference framework, this value represents an internal, selection-informed estimate rather than an independent measure of specificity. Overall, the results indicate that the panel provides a compact resource for studying Kazakh-related genetic ancestry within the tested reference framework.

Previous studies have established the broad East–West Eurasian admixture background of Kazakh populations [1,5,6,7,8]. Consistent with these findings, our PCA and pairwise F_ST_ analyses placed the Kazakh cohort within the broader Central Asian and Inner Eurasian genetic continuum and showed particularly low differentiation from several neighboring populations. This pattern was also reflected in the assignment analysis: the panel transferred well to the two independent Kazakh cohorts, whereas Kazakh-related assignments among non-Kazakh individuals occurred mainly in genetically close Central Asian and Inner Eurasian groups. These findings indicate that the panel captures a Kazakh-related ancestry profile within the tested reference framework rather than genetic markers unique to Kazakh ethnicity.

The marker-selection strategy combined allele-frequency differentiation with informativeness for assignment because both the magnitude and consistency of population differentiation are relevant for AIM discovery. Informativeness for assignment provides an information-theoretic measure of the usefulness of genetic markers for distinguishing ancestry across populations [11]. PCA and model-based population-structure approaches are widely used to characterize genetic structure within reference frameworks [21,22], while previous AIM studies have shown that carefully selected SNP panels can reproduce major ancestry-related patterns using substantially fewer markers than genome-wide datasets [23,24]. In the present study, combining differentiation and informativeness allowed markers to be prioritized according to their ability to distinguish the Kazakh cohort across multiple reference populations rather than against only a single comparison group. The resulting LD-pruned panel therefore reflects the continuous and partially shared ancestry patterns characteristic of Central Asian populations [1,5,6].

The robustness analyses showed that the selected panel was generally stable across alternative analytical settings. Moderate changes in component weighting, removal of the Q10 term, and alternative LD settings retained a substantial proportion of the original markers, whereas more extreme weighting schemes produced greater changes in panel composition. The LD-filtered panel composition was also highly stable when LD was estimated using the Kazakh cohort alone: 383 of the original 385 markers were retained, with a Jaccard similarity of 0.977. In addition, the final panel showed higher marker-level differentiation and informativeness than chromosome-matched random LD-pruned panels, supporting the effectiveness of the marker-selection procedure. The reduced top-200 panel produced lower Kazakh-related assignment rates. Together, these findings suggest that the larger panel provides more stable and informative discrimination within the tested reference framework.

Transferability was evaluated using two independent Kazakh cohorts [6,9]. Because marker availability differed across datasets, the assessment was based on the 341 AIMs shared across the discovery dataset and both external cohorts. Both external cohorts showed substantial overlap with the internal Kazakh cohort in the AIM-based PCA and high Kazakh-related assignment rates in the nearest-centroid analysis. These results support the transferability of the shared marker subset across independent Kazakh datasets. However, because both external datasets consisted of Kazakh individuals, they provide evidence of transferability rather than independent external specificity against non-Kazakh populations.

Kazakh-related assignment rates were 92.0% in the Serikzhan et al. cohort and 91.7% in the Lei et al. cohort. In contrast, 4.1% of the non-Kazakh reference individuals were assigned as Kazakh-related. Because these individuals were part of the discovery and reference framework, this value represents an internal, selection-informed estimate rather than an independent measure of specificity. Population-specific results showed that these assignments occurred mainly among genetically close Central Asian and Inner Eurasian populations, particularly Karakalpak and Kyrgyz individuals, consistent with the very low pairwise F_ST_ values observed between these populations and the Kazakh cohort. The panel therefore captures a genetic profile strongly represented in Kazakhs but partially shared with neighboring populations and has limited ability to make categorical distinctions between Kazakhs and closely related Central Asian populations. This interpretation is consistent with broader evidence that human genetic variation is structured but substantially shared across populations [25,26].

The final Kazakh AIM panel may be useful for several research applications. Compact AIM panels have previously been used to infer ancestry, characterize population structure, and help control for population stratification in genetic association studies [23,24,27,28,29]. In the Kazakh context, the present panel could support studies requiring a focused set of markers for Kazakh-related ancestry assessment, comparative analyses of Kazakh and neighboring populations, or sample-level quality control when genome-wide SNP data are unavailable or unnecessary. However, the panel should not be used as a stand-alone test of ethnic identity, because genetic ancestry inferred from genomic data is distinct from self-identified ethnicity and broader sociocultural identity, including language and culture [30].

Several limitations should be considered. First, the AIM panel was developed and evaluated within a specific reference-population framework, and its performance may change if additional Central Asian, Siberian, West Eurasian, or East Eurasian populations are included. Second, some reference groups had small sample sizes and were excluded from the main AIM-discovery comparisons, although their F_ST_ values were retained in the supplementary analyses. Third, the 624 Kazakh individuals were analyzed as a single target cohort, and the study did not address regional, tribal, or subethnic structure within the Kazakh population. Such fine-scale structure may require larger geographically balanced datasets and more detailed metadata. Fourth, external transferability was evaluated using the 341 AIMs shared across all datasets rather than the complete 385-SNP panel because marker availability differed among genotyping platforms. The external results therefore apply specifically to the shared 341-SNP subset.

Despite these limitations, the present study provides a data-driven Kazakh AIM resource evaluated in two independent Kazakh cohorts. The results show that a compact set of LD-pruned SNPs can preserve major Kazakh-related population structure and transfer across independent Kazakh datasets within the tested reference framework. At the same time, Kazakh-related assignments among non-Kazakh reference individuals were concentrated mainly in genetically close Central Asian and Inner Eurasian populations, emphasizing that the panel captures relative genetic ancestry rather than a population-specific ethnic signature. Overall, the panel represents a useful resource for future population-genetic and genomic studies involving Kazakh-related ancestry, but its performance remains dependent on the reference populations included and it should not be used as a stand-alone test of ethnic identity [30].

## 5. Conclusions

This study identified a 385-SNP ancestry-informative marker panel for evaluating Kazakh-related genetic ancestry within a defined global and Eurasian reference framework. The selected markers preserved the main population-structure patterns observed in the genome-wide dataset while providing a substantially reduced marker set.

Transferability assessment using the 341 markers shared across all datasets produced Kazakh-related assignment rates of 92.0% in the Serikzhan et al. cohort and 91.7% in the Lei et al. cohort. Among non-Kazakh reference individuals, the internal, selection-informed assignment rate was 4.1%, with most Kazakh-related assignments occurring in genetically close Central Asian and Inner Eurasian populations. These findings support the transferability of the shared marker subset across independent Kazakh datasets while highlighting reduced discrimination between Kazakhs and closely related neighboring populations.

## Figures and Tables

**Table 1:** Composition of the discovery dataset used for Kazakh AIM discovery.

Dataset/Source	Population Groups	Individuals	Role
Kazakh cohort from the present study	1	624	Target population
EGDP	29	433	Regional reference panel
1000 Genomes Project	11	550	Global reference panel
Total	41	1607	Discovery dataset

Note: EGDP, Estonian Biocentre Human Genome Diversity Panel. The discovery dataset included the target Kazakh cohort and external reference populations.

**Table 2:** Nearest-centroid assignment results using the 341 AIMs shared across all datasets.

Assessment Group	Dataset Role	Assigned as Kazakh- Related (n/N)	Kazakh-Related Assignment Rate, % (95% CI)
Internal Kazakh cohort	Internal leave-one-out assessment	590/624	94.6 (92.5–96.1)
Serikzhan et al. cohort [9]	Independent Kazakh transferability assessment	206/224	92.0 (87.7–94.9)
Lei et al. cohort [6]	Independent Kazakh transferability assessment	121/132	91.7 (85.7–95.3)
Non-Kazakh reference individuals	Internal, selection-informed non-Kazakh assignment assessment	38/934	4.1 (3.0–5.5)

Note: Results are reported as the number assigned as Kazakh-related relative to the total number evaluated (n/N), together with the corresponding percentage and 95% Wilson confidence interval. Internal Kazakh and non-Kazakh reference individuals were evaluated using a leave-one-out centroid procedure. Missing genotype values were replaced with SNP-specific mean dosages calculated exclusively from the internal Kazakh and non-Kazakh reference samples. The external Serikzhan et al. [9] and Lei et al. [6] cohorts were not used for missing-genotype replacement, centroid construction, or definition of the assignment rule. Because the non-Kazakh reference individuals contributed to the discovery/reference framework, their 4.1% Kazakh-related assignment rate is interpreted as an internal, selection-informed estimate rather than as an independent external specificity estimate.

## Data Availability

Individual-level genotype data from the internal Kazakh cohort are not publicly available because of ethical and privacy restrictions related to human genetic data. Additional information concerning the internal dataset may be made available from the corresponding author upon reasonable request and subject to institutional and ethical approvals. The EGDP reference data were obtained from the Estonian Biocentre public data resource associated with Pagani et al. [17]. Selected 1000 Genomes Project Phase 3 reference data were obtained from the 1000 Genomes Phase 3 reference dataset distributed through the PLINK 2.0 Resources repository. The Serikzhan et al. external Kazakh dataset was obtained from the European Variation Archive under accession PRJEB89820. The Lei et al. external Kazakh genotype dataset was obtained through controlled access from the Genome Variation Map under project accession GVM000484. A reproducibility archive (Supplementary_File_S1_Reproducibility_Archive.zip) containing analysis scripts, command-line workflows, software-environment information, marker-level intermediate results, and non-identifying population-level allele-frequency data is provided as Supplementary File S1.

## Supplementary Materials

The following supporting information can be downloaded at: https://www.mdpi.com/article/10.3390/life16101652/s1, Figure S1: Eurasian principal component analysis of the Kazakh cohort and selected reference populations; Table S1: Full composition of the discovery dataset; Table S2: Pairwise Weir–Cockerham FST values between the Kazakh cohort and global reference populations; Table S3: Pairwise Weir–Cockerham FST values between the Kazakh cohort and Eurasian reference populations; Table S4: Pairwise Weir–Cockerham FST values between the Kazakh cohort and all fine-scale reference populations, including populations represented by fewer than 10 individuals; Table S5: Pairwise Weir–Cockerham FST values used for the main fine-scale analysis, restricted to reference populations represented by at least 10 individuals; Table S6: Final 385-SNP fine-scale Kazakh AIM panel with marker-selection metrics, PCA loading support, and Ensembl VEP genomic annotations; Table S7: Robustness analyses of AIM selection under alternative ranking, candidate-pool, LD, and panel-size settings; Table S8: LD-excluded candidate SNPs and corresponding retained higher-ranked markers, including pairwise r2 values and genomic distances; Table S9: Benchmark comparison of the final 385-SNP panel with 100 chromosome-matched random LD-pruned panels; Table S10: Marker overlap between the final 385-SNP AIM panel and the two independent external Kazakh cohorts; Table S11: Nearest-centroid assignment results by cohort and non-Kazakh reference population using the 341 AIMs shared across all datasets; Table S12: Stepwise summary of marker retention during dataset harmonization and external-panel matching; File S1: Supplementary_File_S1_Reproducibility_Archive.zip, containing analysis scripts, command-line workflows, software-environment information, marker-level intermediate results, and non-identifying population-level allele-frequency data.

## Abbreviations

The following abbreviations are used in this manuscript:

AIM	Ancestry-informative marker
DNA	Deoxyribonucleic acid
EGDP	Estonian Biocentre Human Genome Diversity Panel
*F*_ST_	Fixation index
GSA	Global Screening Array
LD	Linkage disequilibrium
MXL	Mexican Ancestry from Los Angeles, California
PC	Principal component
PCA	Principal component analysis
SNP	Single-nucleotide polymorphism
