# Supplementary material for: Genome-Wide Discovery and Evaluation of Kazakh Ancestry-Informative Markers
Source: Life (Basel). 2026 Sep 30;16(10):1652. doi: 10.3390/life16101652 (PMC13705973; doi:10.3390/life16101652)
Supplement: Supplementary file 1 [file life-16-01652-s001.zip › Supplementary_files/Supplementary_Tables_Kazakh_AIM_FINAL/FigureS1_eurasian_PCA.pdf]

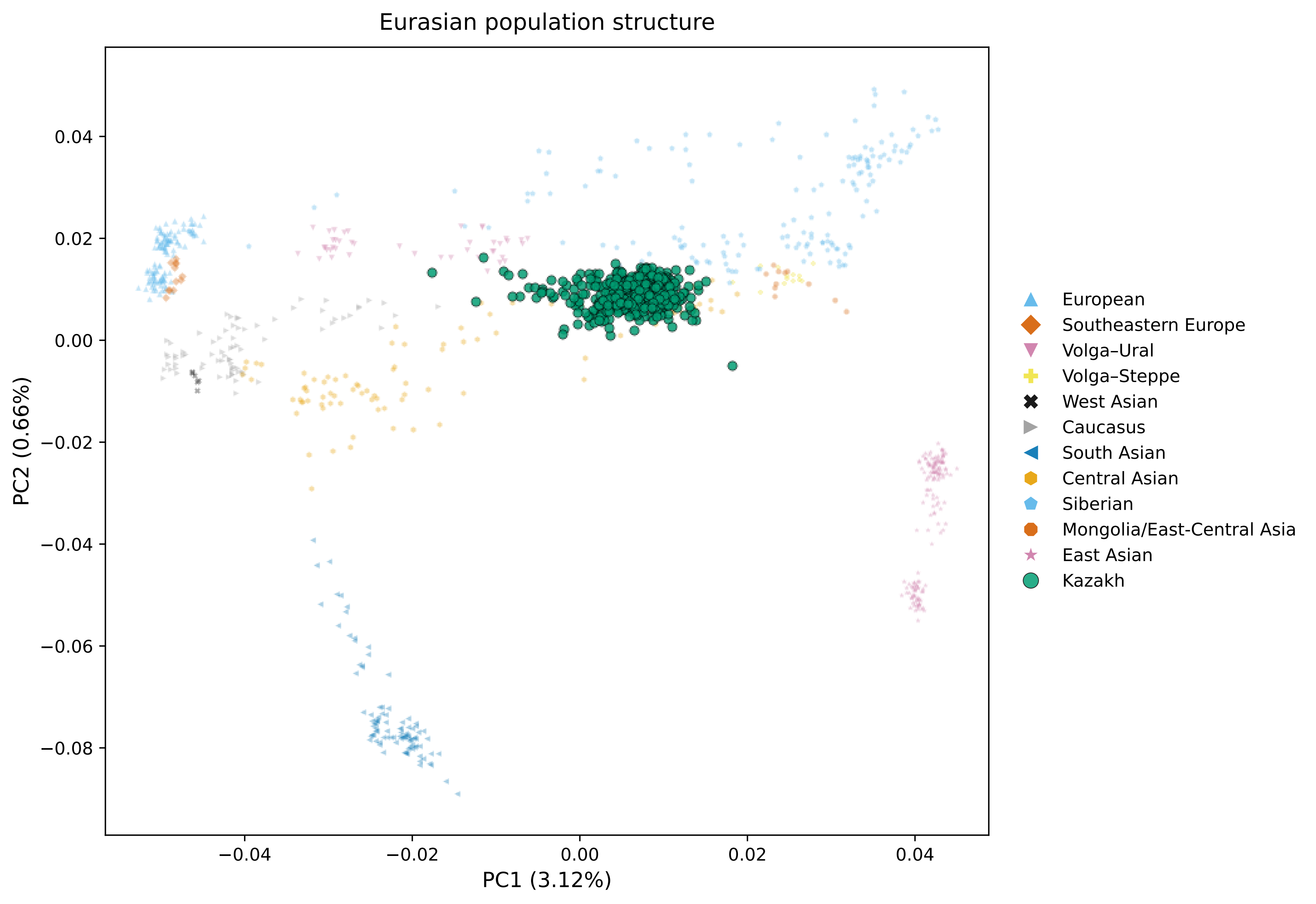

Eurasian population structure
PC1 (3.12%)
PC2 (0.66%)
−0.04
−0.02
0.00
0.02
0.04
0.04
0.02
0.00
−0.02
−0.04
−0.06
−0.08
European
Southeastern Europe
Volga–Ural
Volga–Steppe
West Asian
Caucasus
South Asian
Central Asian
Siberian
Mongolia/East-Central Asia
East Asian
Kazakh
